# Supplementary material for: gmxapi: A GROMACS-native Python interface for molecular dynamics with ensemble and plugin support
Source: PLoS Comput Biol. 2022 Feb 14;18(2):e1009835. doi: 10.1371/journal.pcbi.1009835 (PMC8880871; doi:10.1371/journal.pcbi.1009835)
Supplement: S1 Software Archive — (TGZ) [file pcbi.1009835.s002.tgz › 2022-software/run-brer-master.tar.gz!/run_brer-master/docs/_build/html/developer_api.html]

Developer API — run BRER 1.0.0 documentation


run BRER

Contents:

- User API
- Developer API
  - directory\_helper
  - metadata
  - pair\_data
  - plugin\_configs

run BRER

- »
- Developer API
- View page source

---

# Developer API¶

## directory\_helper¶

Organizes the directory structure for BRER runs. Creates directories on the
fly.

How the directory structure is organized:
:   - This script should be run from your “top” directory (where
      you are planning to run all your ensemble members)
    - The top directory contains ensemble member subdirectories
      This script is intended to handle just ONE ensemble member,
      so we’ll only be concerned with a single member subdirectory.
    - The example below is for the first iteration (iter 0) of one
      of the members. Future iterations would go in directories
      1,2,…y

*class* `run_brer.directory_helper.``DirectoryHelper`(*top\_dir*, *param\_dict*)¶
:   Small class for manipulating a standard directory structure for BRER
    runs.

    Parameters
    :   - **top\_dir** – the path to the directory containing all the ensemble members.
        - **param\_dict** – a dictionary specifying the ensemble number, the iteration,
          and the phase of the simulation.

    Examples

    ```
    >>> .
    >>> ├── 0
    >>> │   ├── converge_dist
    >>> │   │   ├── state.cpt
    >>> │   │   ├── state_prev.cpt
    >>> │   │   └── traj_comp.part0001.xtc
    >>> │   ├── production
    >>> │   │   ├── confout.part0005.gro
    >>> │   │   ├── state.cpt
    >>> │   │   ├── state_prev.cpt
    >>> │   │   ├── state_step4622560.cpt
    >>> │   │   ├── traj_comp.part0002.xtc
    >>> │   └── training
    >>> │       ├── state.cpt
    >>> │       ├── state_prev.cpt
    >>> │       └── traj_comp.part0001.xtc
    >>> ├── state.json
    >>> ├── submit.slurm
    >>> └── syx.tpr
    ```

    `build_working_dir`()¶
    :   Checks to see if the working directory for current state of BRER
        simulation exists. If it does not, creates the directory.

    `change_dir`(*level*)¶
    :   Change to directory specified by level.

        Parameters
        :   **level** (*str*) – How far to go down the directory tree.
            Can be one of ‘top’, ‘ensemble\_num’, ‘iteration’, or ‘phase’.

    `get_dir`(*level*)¶
    :   Get the directory for however far you want to go down the directory
        tree.

        Parameters
        :   **level** – one of ‘top’, ‘ensemble\_num’, ‘iteration’, or ‘phase’.
            See the directory structure example provided at the beginning of this class.

        Returns
        :   the path to the specified directory ‘level’ as a str.

        Return type
        :   type

## metadata¶

Abstract class for handling all BRER metadata.

State and PairData classes inherit from this class.

*class* `run_brer.metadata.``MetaData`(*name*)¶
:   Construct metadata object. and give it a name.

    Parameters
    :   **name** – Give your MetaData class a descriptive name.

    `get`(*key*)¶
    :   Get the value of a parameter of the class.

        Parameters
        :   **key** (*str*) – The name of the parameter you wish to get.

        Returns
        :   The value of the parameter associated with the key.

        Return type
        :   type

    `get_as_dictionary`()¶
    :   Get all of the current parameters of the class as a dictionary.

        Returns
        :   dictionary of class parameters

        Return type
        :   dict

    `get_missing_keys`()¶
    :   Gets all of the required parameters that have not been set.

        Returns
        :   A list of required parameter names that have not been set.

        Return type
        :   list

    `get_requirements`()¶
    :   Gets the set of required parameters for the class. This is quite
        useful for checking if there are any missing parameters before
        beginning a run.

        Returns
        :   required parameters of the class

        Return type
        :   list

    *property* `name`¶
    :   getter for name.

        Returns


        Return type
        :   str

    `set`(*key=None*, *value=None*, *\*\*kwargs*)¶
    :   Sets a parameter of the class. Checks whether or not the parameter
        is required and reports information about requirements. You can pass
        the key,value pairs either as a key and value or as a set of \*\*kwargs.

        Parameters
        :   - **key** (*str**,* *optional*) – parameter name, by default None
            - **value** (*any**,* *optional*) – parameter value, by default None

    `set_from_dictionary`(*data: dict*)¶
    :   Another method that essentially performs the same thing as “set”,
        but just takes a dictionary. Probably should be deprecated…

        Parameters
        :   **data** (*dict*) – A dictionary of parameter names and values to set.

    `set_requirements`(*list\_of\_requirements: list*)¶
    :   Defines a set of required parameters for the class. This is quite
        useful for checking if there are any missing parameters before
        beginning a run.

        Parameters
        :   **list\_of\_requirements** (*list*) – list of required parameters for the class (a list of strings)

*class* `run_brer.metadata.``MultiMetaData`¶
:   A single class that handles multiple MetaData classes (useful when
    restraining multiple atom-atom pairs).

    `add_metadata`(*metadata: run\_brer.metadata.MetaData*)¶
    :   Appends new MetaData object to self.\_metadata.

        Parameters
        :   **metadata** (*MetaData*) – metadata to append

    `id_to_name`(*id*)¶
    :   Converts the index of one of the MetaData classes to it’s associated
        name.

        Parameters
        :   **id** (*int*) – The index of the MetaData class in the list self.\_metadata

        Returns
        :   the name of the metadata class

        Return type
        :   str

    `name_to_id`(*name*)¶
    :   Converts the name of one of the MetaData classes to it’s associated
        list index (it’s idx in self.\_metadata)

        Parameters
        :   **name** (*str*) – the name of a MetaData class.

        Returns
        :   the index of the MetaData class in the self.\_metadata list.

        Return type
        :   int

        Raises
        :   **IndexError** – Raise IndexError if no metadata have been loaded.

    *property* `names`¶
    :   getter for names.

        Returns
        :   list of names

        Return type
        :   list

        Raises
        :   **IndexError** – Raise IndexError if no metadata have been loaded.

    `read_from_json`(*filename='state.json'*)¶
    :   Reads state from json.

        Parameters
        :   **filename** (*str*) – (Default value = ‘state.json’)

    `write_to_json`(*filename='state.json'*)¶
    :   Writes state to json.

        Parameters
        :   **filename** (*str*) – (Default value = ‘state.json’)

## pair\_data¶

Classes to handle 1) pair metadata 2) resampling from DEER distributions at
each new BRER iteration.

*class* `run_brer.pair_data.``PairData`(*name*)¶
:   Class to handle pair metadata (distribution, bins, atom ids)

    Construct metadata object. and give it a name.

    Parameters
    :   **name** – Give your MetaData class a descriptive name.

*class* `run_brer.pair_data.``MultiPair`¶
:   Single class for handling multiple pair data.

    Handles resampling of targets.

    `re_sample`()¶
    :   Re-sample from the joint space. Do normalization just in case the
        data aren’t normalized already.

        Returns
        :   dictionary of targets, drawn from DEER distributions.

        Return type
        :   dict

    `read_from_json`(*filename='state.json'*)¶
    :   Reads pair data from json file. For an example file, see
        pair\_data.json in the data directory.

        Parameters
        :   **filename** (*str**,* *optional*) – filename of the pair data, by default ‘state.json’

## plugin\_configs¶

Classes used to build gmxapi plugins for all phases of a BRER iteration Each
class corresponds to ONE restraint since gmxapi plugins each correspond to one
restraint.

*class* `run_brer.plugin_configs.``PluginConfig`¶
:   Abstract class used to build training, convergence, and production
    plugins.

    Construct metadata object. and give it a name.

    Parameters
    :   **name** – Give your MetaData class a descriptive name.

    *abstract* `build_plugin`()¶
    :   Abstract method for building a plugin.

        To be determined by the phase of the simulation (training,
        convergence, production)

    `scan_dictionary`(*dictionary*)¶
    :   Scans a dictionary and stores whatever parameters it needs for the
        build\_plugin.

        Parameters
        :   **dictionary** (*dict*) – a dictionary containing metadata, some of which may be needed for the run.
            The dictionary may contain *extra* data, i.e., this can be a superset of the
            needed plugin data.

    `scan_metadata`(*data*)¶
    :   This scans a RunData or PairData obj and stores whatever parameters
        it needs for a run.

        Parameters
        :   **data** – either type RunData or type PairData

*class* `run_brer.plugin_configs.``TrainingPluginConfig`¶
:   Construct metadata object. and give it a name.

    Parameters
    :   **name** – Give your MetaData class a descriptive name.

    `build_plugin`()¶
    :   Builds training phase plugin for BRER simulations.

        Returns
        :   a gmxapi WorkElement to be added to the workflow graph

        Return type
        :   WorkElement

        Raises
        :   **KeyError** – if required parameters for building the plugin are missing.

*class* `run_brer.plugin_configs.``ConvergencePluginConfig`¶
:   Construct metadata object. and give it a name.

    Parameters
    :   **name** – Give your MetaData class a descriptive name.

    `build_plugin`()¶
    :   Builds convergence phase plugin for BRER simulations.

        Returns
        :   a gmxapi WorkElement to be added to the workflow graph

        Return type
        :   WorkElement

        Raises
        :   **KeyError** – if required parameters for building the plugin are missing.

*class* `run_brer.plugin_configs.``ProductionPluginConfig`¶
:   Construct metadata object. and give it a name.

    Parameters
    :   **name** – Give your MetaData class a descriptive name.

    `build_plugin`()¶
    :   Builds production phase plugin for BRER simulations.

        Returns
        :   a gmxapi WorkElement to be added to the workflow graph

        Return type
        :   WorkElement

        Raises
        :   **KeyError** – if required parameters for building the plugin are missing.

Previous

---

© Copyright 2019, Jennifer M. Hays

Built with Sphinx using a
theme
provided by Read the Docs.
