# Supplementary material for: gmxapi: A GROMACS-native Python interface for molecular dynamics with ensemble and plugin support
Source: PLoS Comput Biol. 2022 Feb 14;18(2):e1009835. doi: 10.1371/journal.pcbi.1009835 (PMC8880871; doi:10.1371/journal.pcbi.1009835)
Supplement: S1 Software Archive — (TGZ) [file pcbi.1009835.s002.tgz › 2022-software/run-brer-master.tar.gz!/run_brer-master/docs/_build/html/genindex.html]

Index — run BRER 1.0.0 documentation


run BRER

Contents:

- User API
- Developer API

run BRER

- »
- Index

---

# Index

**A**
| **B**
| **C**
| **D**
| **F**
| **G**
| **I**
| **L**
| **M**
| **N**
| **P**
| **R**
| **S**
| **T**
| **W**

## A

|  |  |
| --- | --- |
| - add\_metadata() (run\_brer.metadata.MultiMetaData method) | - as\_dictionary() (run\_brer.run\_data.RunData method) |

## B

|  |  |
| --- | --- |
| - build\_plugin() (run\_brer.plugin\_configs.ConvergencePluginConfig method)   - (run\_brer.plugin\_configs.PluginConfig method)   - (run\_brer.plugin\_configs.ProductionPluginConfig method)   - (run\_brer.plugin\_configs.TrainingPluginConfig method) | - build\_plugins() (run\_brer.run\_config.RunConfig method) - build\_working\_dir() (run\_brer.directory\_helper.DirectoryHelper method) |

## C

|  |  |
| --- | --- |
| - change\_dir() (run\_brer.directory\_helper.DirectoryHelper method) | - clear\_pair\_data() (run\_brer.run\_data.RunData method) - ConvergencePluginConfig (class in run\_brer.plugin\_configs) |

## D

- DirectoryHelper (class in run\_brer.directory\_helper)

## F

|  |  |
| --- | --- |
| - from\_dictionary() (run\_brer.run\_data.RunData method) | - from\_pair\_data() (run\_brer.run\_data.RunData method) |

## G

|  |  |
| --- | --- |
| - get() (run\_brer.metadata.MetaData method)   - (run\_brer.run\_data.RunData method) - get\_as\_dictionary() (run\_brer.metadata.MetaData method) | - get\_dir() (run\_brer.directory\_helper.DirectoryHelper method) - get\_missing\_keys() (run\_brer.metadata.MetaData method) - get\_requirements() (run\_brer.metadata.MetaData method) |

## I

- id\_to\_name() (run\_brer.metadata.MultiMetaData method)

## L

- load\_config() (run\_brer.run\_data.RunData method)

## M

|  |  |
| --- | --- |
| - MetaData (class in run\_brer.metadata) - module   - run\_brer.directory\_helper   - run\_brer.metadata   - run\_brer.pair\_data   - run\_brer.plugin\_configs   - run\_brer.run\_config   - run\_brer.run\_data | - MultiMetaData (class in run\_brer.metadata) - MultiPair (class in run\_brer.pair\_data) |

## N

|  |  |
| --- | --- |
| - name() (run\_brer.metadata.MetaData property) | - name\_to\_id() (run\_brer.metadata.MultiMetaData method) - names() (run\_brer.metadata.MultiMetaData property) |

## P

|  |  |
| --- | --- |
| - PairData (class in run\_brer.pair\_data) | - PluginConfig (class in run\_brer.plugin\_configs) - ProductionPluginConfig (class in run\_brer.plugin\_configs) |

## R

|  |  |
| --- | --- |
| - re\_sample() (run\_brer.pair\_data.MultiPair method) - read\_from\_json() (run\_brer.metadata.MultiMetaData method)   - (run\_brer.pair\_data.MultiPair method) - run() (run\_brer.run\_config.RunConfig method) - run\_brer.directory\_helper   - module - run\_brer.metadata   - module - run\_brer.pair\_data   - module | - run\_brer.plugin\_configs   - module - run\_brer.run\_config   - module - run\_brer.run\_data   - module - RunConfig (class in run\_brer.run\_config) - RunData (class in run\_brer.run\_data) |

## S

|  |  |
| --- | --- |
| - save\_config() (run\_brer.run\_data.RunData method) - scan\_dictionary() (run\_brer.plugin\_configs.PluginConfig method) - scan\_metadata() (run\_brer.plugin\_configs.PluginConfig method) | - set() (run\_brer.metadata.MetaData method)   - (run\_brer.run\_data.RunData method) - set\_from\_dictionary() (run\_brer.metadata.MetaData method) - set\_requirements() (run\_brer.metadata.MetaData method) |

## T

- TrainingPluginConfig (class in run\_brer.plugin\_configs)

## W

- write\_to\_json() (run\_brer.metadata.MultiMetaData method)

---

© Copyright 2019, Jennifer M. Hays

Built with Sphinx using a
theme
provided by Read the Docs.
