# Supplementary material for: gmxapi: A GROMACS-native Python interface for molecular dynamics with ensemble and plugin support
Source: PLoS Comput Biol. 2022 Feb 14;18(2):e1009835. doi: 10.1371/journal.pcbi.1009835 (PMC8880871; doi:10.1371/journal.pcbi.1009835)
Supplement: S1 Software Archive — (TGZ) [file pcbi.1009835.s002.tgz › 2022-software/run-brer-master.tar.gz!/run_brer-master/docs/_build/html/index.html]

Welcome to run BRER’s documentation! — run BRER 1.0.0 documentation


run BRER

Contents:

- User API
- Developer API

run BRER

- »
- Welcome to run BRER’s documentation!
- View page source

---

# Welcome to run BRER’s documentation!¶

Contents:

- User API
  - run\_brer
  - run\_config
- Developer API
  - directory\_helper
  - metadata
  - pair\_data
  - plugin\_configs

# run\_brer¶

Set of scripts for running BRER simulations using gmxapi. Details of
this method may be found at:

Hays, J. M., Cafiso, D. S., & Kasson, P. M. Hybrid Refinement of
Heterogeneous Conformational Ensembles using Spectroscopic Data. *The
Journal of Physical Chemistry Letters*. DOI:
10.1021/acs.jpclett.9b01407

## Installation¶

### Requirements¶

If you’re going to use a pip or a conda environment, you’ll need:

- Python 3.X
- gmxapi for GROMACS 2019. See recommended
  installation,
  or

  1. Install GROMACS
     2019,
     configured with
     `` `-DGMXAPI=ON `` <http://manual.gromacs.org/2019-current/dev-manual/build-system.html#cmake-GMXAPI>`\_\_.
     Currently, `gmxapi` does not support domain decomposition with
     MPI, so if you want these simulations to run fast, be sure to
     compile with GPU support.
  2. Install gmxapi 0.0.7 Python
     package.
     This code has only been tested with Gromacs
     2019.
- The plugin
  code for
  BRER. Please make sure you install the `brer` branch, *NOT*
  `master` .

Otherwise, you can just use a Singularity container!

### Singularity¶

By far the easiest option!

If you have the latest and greatest Singuarity (v > 3), you can pull the
container from the cloud repository:

`singularity pull library://kassonlab/default/brer:latest`

For instructions on using the container, please see the
singularity-brer
repository.

### Conda environment¶

I suggest running this in a conda environment rather than
`pip install` . The following conda command will handle all the
`gmxapi` and `sample_restraint` python dependencies, as well as the
ones for this repository.

1. `conda create -n BRER numpy scipy networkx setuptools mpi4py cmake`

   If you want to run the tests, then install `pytest` as well.
2. Source the environment and then `pip install`:

```
source activate BRER
git clone https://github.com/kassonlab/run_brer.git
cd run_brer
pip install .
```

## Running BRER¶

An example script, `run.py` , is provided for ensemble simulations.

Let’s work through it piece by piece.

```
#!/usr/bin/env python

"""
Example run script
for BRER simulations
"""

import run_brer.run_config as rc
import sys
```

The `import run_brer.run_config` statement imports a `RunConfig`
object, which handles the following things **for a single ensemble
member**:

1. Initializing/setting up parameters for the BRER run.
2. Launching the run.

Then we provide some files and directory paths to the `RunConfig`
object.

```
init = {
    'tpr': '/home/jennifer/Git/run_brer/tests/syx.tpr',
    'ensemble_dir': '/home/jennifer/test-brer',
    'ensemble_num': 5,
    'pairs_json': '/home/jennifer/Git/run_brer/tests/pair_data.json'
}

config = rc.RunConfig(**init)
```

In order to run a BRER simulation, we need to provide :

1. a `tpr` (compatible with GROMACS 2019).
2. The path to our ensemble. This directory should contain
   subdirectories of the form `mem_<my ensemble number>`
3. The ensemble number. This is an integer used to identify which
   ensemble member we are running and thus, the subdirectory in which we
   will be running our simulations.
4. The path to the DEER metadata. Please see the example json in this
   repository: `run_brer/data/pair_data.json`

Finally, we launch the run!

```
config.run()
```

You may change various parameters before launching the run using
`config.set(**kwargs)` . For example:

```
config = rc.RunConfig(**init)
config.set(A=100)
config.run()
```

resets the energy constant A to 100 kcal/mol/nm^2 before launching a
run.

Right now, the way to launch an ensemble is to launch multiple jobs. We
hope to soon use the `gmxapi`
features that allow a user to
launch many ensemble members in one job.

# Indices and tables¶

- Index
- Module Index
- Search Page

Next

---

© Copyright 2019, Jennifer M. Hays

Built with Sphinx using a
theme
provided by Read the Docs.
