# Supplementary material for: gmxapi: A GROMACS-native Python interface for molecular dynamics with ensemble and plugin support
Source: PLoS Comput Biol. 2022 Feb 14;18(2):e1009835. doi: 10.1371/journal.pcbi.1009835 (PMC8880871; doi:10.1371/journal.pcbi.1009835)
Supplement: S1 Software Archive — (TGZ) [file pcbi.1009835.s002.tgz › 2022-software/run-brer-master.tar.gz!/run_brer-master/docs/_build/html/search.html]

Search — run BRER 1.0.0 documentation


run BRER

Contents:

- User API
- Developer API

run BRER

- »
- Search

---

Please activate JavaScript to enable the search functionality.

---

© Copyright 2019, Jennifer M. Hays

Built with Sphinx using a
theme
provided by Read the Docs.
