# Supplementary material for: gmxapi: A GROMACS-native Python interface for molecular dynamics with ensemble and plugin support
Source: PLoS Comput Biol. 2022 Feb 14;18(2):e1009835. doi: 10.1371/journal.pcbi.1009835 (PMC8880871; doi:10.1371/journal.pcbi.1009835)
Supplement: S1 Software Archive — (TGZ) [file pcbi.1009835.s002.tgz › 2022-software/run-brer-master.tar.gz!/run_brer-master/docs/_build/html/user_api.html]

User API — run BRER 1.0.0 documentation


run BRER

Contents:

- User API
  - run\_brer
  - run\_config
- Developer API

run BRER

- »
- User API
- View page source

---

# User API¶

## run\_brer¶

RunConfig class handles the actual workflow logic.

*class* `run_brer.run_config.``RunConfig`(*tpr*, *ensemble\_dir*, *ensemble\_num=1*, *pairs\_json='pair\_data.json'*)¶
:   Run configuration for single BRER ensemble member.

    The run configuration specifies the files and directory structure
    used for the run. It determines whether the run is in the training,
    convergence, or production phase, then performs the run.

    Parameters
    :   - **tpr** (*str*) – path to tpr. Must be gmx 2017 compatible.
        - **ensemble\_dir** (*str*) – path to top directory which contains the full ensemble.
        - **ensemble\_num** (*int**,* *optional*) – the ensemble member to run, by default 1
        - **pairs\_json** (*str**,* *optional*) – path to file containing *ALL* the pair metadata.
          An example of what such a file should look like is provided in the data directory,
          by default ‘pair\_data.json’

    `build_plugins`(*plugin\_config: run\_brer.plugin\_configs.PluginConfig*)¶
    :   Builds the plugin configuration. For each pair-wise restraint,
        populate the plugin with data: both the “general” data and the data
        unique to that restraint.

        Parameters
        :   **plugin\_config** (*PluginConfig*) – the particular plugin configuration (Training, Convergence, Production) for the run.

    `run`()¶
    :   Perform the MD simulations.

        Each Python interpreter process runs a separate ensemble member.

## run\_config¶

Class that handles the simulation data for BRER simulations.

*class* `run_brer.run_data.``RunData`¶
:   Stores (and manipulates, to a lesser extent) all the metadata for a BRER
    run.

    The full set of metadata for a single BRER run include both the
    general parameters and the pair-specific parameters.

    `as_dictionary`()¶
    :   Get the run metadata as a heirarchical dictionary:

        Returns
        :   heirarchical dictionary of metadata

        Return type
        :   type

        Examples

        ```
        >>> ├── pair parameters
        >>> │   ├── name of pair 1
        >>> │   │   ├── alpha
        >>> │   │   ├── target
        >>> │   │   └── ...
        >>> │   ├── name of pair 2
        >>> |
        >>> ├── general parameters
        >>>     ├── A
        >>>     ├── tau
        >>>     ├── ...
        ```

    `clear_pair_data`()¶
    :   Removes all the pair parameters, replace with empty dict.

    `from_dictionary`(*data: dict*)¶
    :   Loads metadata into the class from a dictionary.

        Parameters
        :   **data** (*dict*) – RunData metadata as a dictionary.

    `from_pair_data`(*pd: run\_brer.pair\_data.PairData*)¶
    :   Load some of the run metadata from a PairData object. Useful at the
        beginning of a run.

        Parameters
        :   **pd** (*PairData*) – object from which metadata are loaded

    `get`(*key*, *name=None*)¶
    :   get either a general or a pair-specific parameter.

        Parameters
        :   - **key** (*str*) – the parameter to get.
            - **name** (*str*) – if getting a pair-specific parameter, specify the restraint name. (Default value = None)

        Returns
        :   the parameter value.

        Return type
        :   type

    `load_config`(*fnm='state.json'*)¶
    :   Load state parameters from file.

        Parameters
        :   **fnm** (*str**,* *optional*) – log file of state parameters, by default ‘state.json’

    `save_config`(*fnm='state.json'*)¶
    :   Saves the run parameters to a log file.

        Parameters
        :   **fnm** (*str**,* *optional*) – log file for state parameters, by default ‘state.json’

    `set`(*name=None*, *\*\*kwargs*)¶
    :   method used to set either general or a pair-specific parameter.

        Parameters
        :   **name** (*str**,* *optional*) – restraint name.
            These are the same identifiers that are used in the RunConfig, by default None

        Raises
        :   **ValueError** – if you provide a name and try to set a general parameter or
            don’t provide a name and try to set a pair-specific parameter.

Next 
 Previous

---

© Copyright 2019, Jennifer M. Hays

Built with Sphinx using a
theme
provided by Read the Docs.
