# Supplementary material for: gmxapi: A GROMACS-native Python interface for molecular dynamics with ensemble and plugin support
Source: PLoS Comput Biol. 2022 Feb 14;18(2):e1009835. doi: 10.1371/journal.pcbi.1009835 (PMC8880871; doi:10.1371/journal.pcbi.1009835)
Supplement: S1 Software Archive — (TGZ) [file pcbi.1009835.s002.tgz › 2022-software/gromacs-v2022-beta2.tar.bz2!/gromacs-v2022-beta2/docs/reference-manual/algorithms/plots/flowchart.pdf]

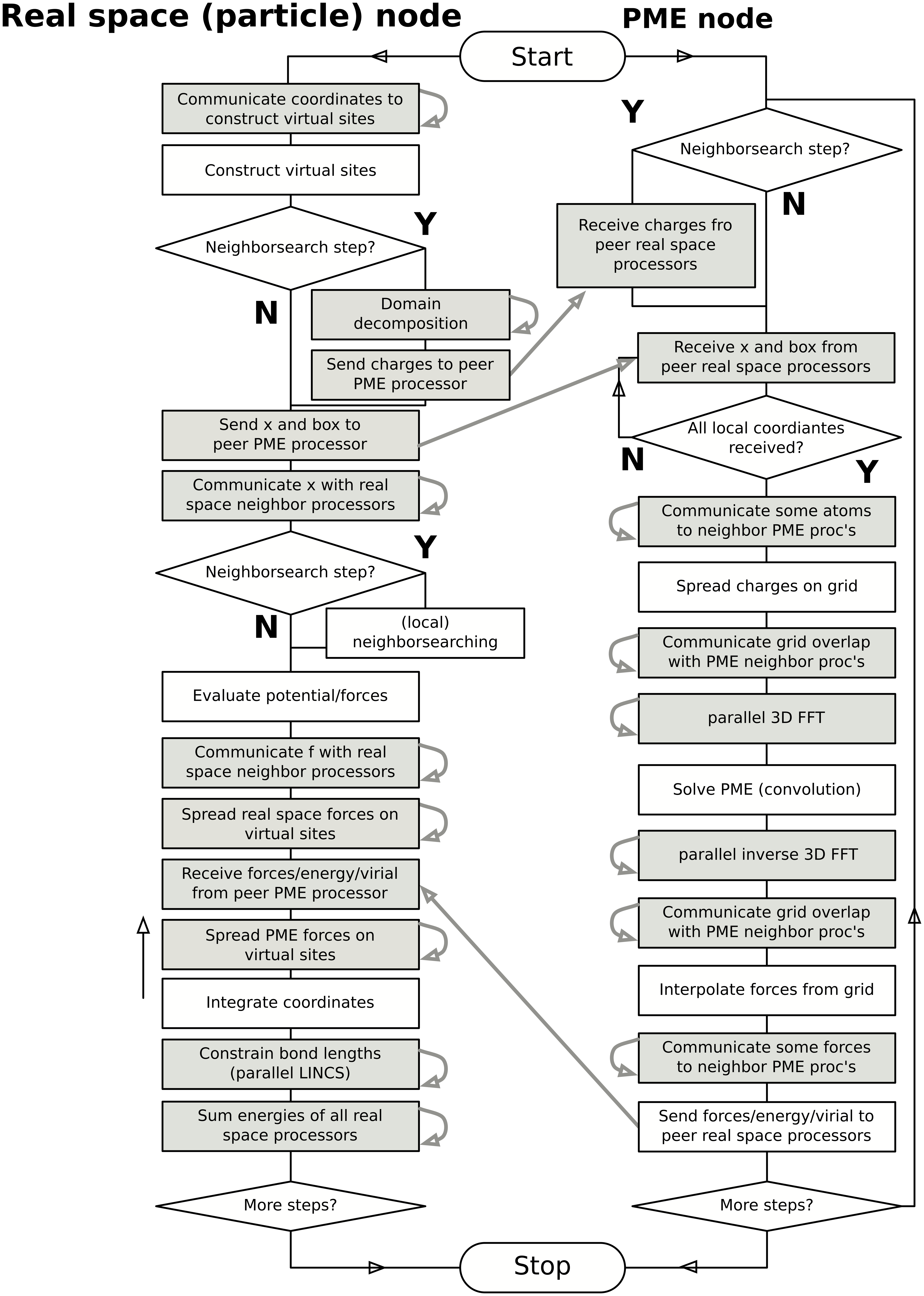

Real space (particle) node
PME node
Start
Communicate coordinates to construct virtual sites
Construct virtual sites
Neighborsearch step?
Y
N
Domain decomposition
Send charges to peer PME processor
Send x and box to peer PME processor
Communicate x with real space neighbor processors
Neighborsearch step?
Y
N
(local) neighborsearching
Evaluate potential/forces
Communicate f with real space neighbor processors
Spread real space forces on virtual sites
Receive forces/energy/virial from peer PME processor
Spread PME forces on virtual sites
Integrate coordinates
Constrain bond lengths (parallel LINCS)
Sum energies of all real space processors
More steps?
Y
Neighborsearch step?
N
Receive charges fro peer real space processors
Receive x and box from peer real space processors
All local coordiantes received?
N
Y
Communicate some atoms to neighbor PME proc's
Spread charges on grid
Communicate grid overlap with PME neighbor proc's
parallel 3D FFT
Solve PME (convolution)
parallel inverse 3D FFT
Communicate grid overlap with PME neighbor proc's
Interpolate forces from grid
Communicate some forces to neighbor PME proc's
Send forces/energy/virial to peer real space processors
More steps?
Stop
