# Supplementary material for: gmxapi: A GROMACS-native Python interface for molecular dynamics with ensemble and plugin support
Source: PLoS Comput Biol. 2022 Feb 14;18(2):e1009835. doi: 10.1371/journal.pcbi.1009835 (PMC8880871; doi:10.1371/journal.pcbi.1009835)
Supplement: S1 Software Archive — (TGZ) [file pcbi.1009835.s002.tgz › 2022-software/gromacs-v2022-beta2.tar.bz2!/gromacs-v2022-beta2/docs/reference-manual/algorithms/plots/verlet-drift.pdf]

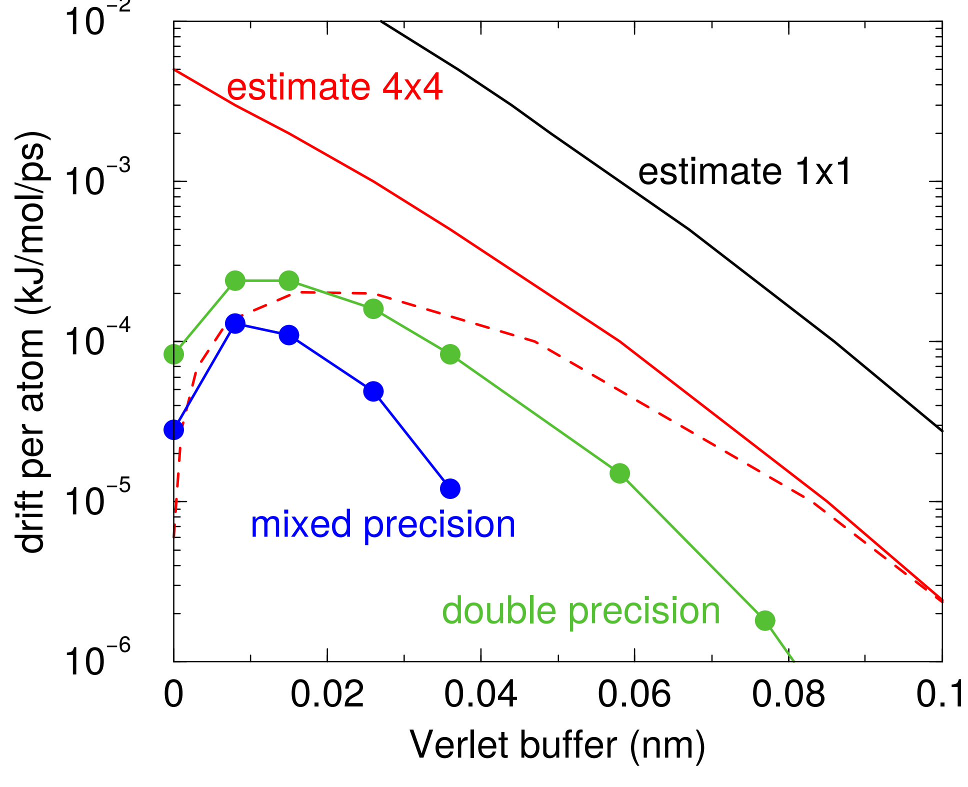

estimate 4x4
estimate 1x1
mixed precision
double precision
drift per atom (kJ/mol/ps)
Verlet buffer (nm)
10^{-2}
10^{-3}
10^{-4}
10^{-5}
10^{-6}
0
0.02
0.04
0.06
0.08
0.1
