# Supplementary material for: gmxapi: A GROMACS-native Python interface for molecular dynamics with ensemble and plugin support
Source: PLoS Comput Biol. 2022 Feb 14;18(2):e1009835. doi: 10.1371/journal.pcbi.1009835 (PMC8880871; doi:10.1371/journal.pcbi.1009835)
Supplement: S1 Software Archive — (TGZ) [file pcbi.1009835.s002.tgz › 2022-software/gromacs-v2022-beta2.tar.bz2!/gromacs-v2022-beta2/docs/reference-manual/analysis/plots/hpr-wheel.pdf]

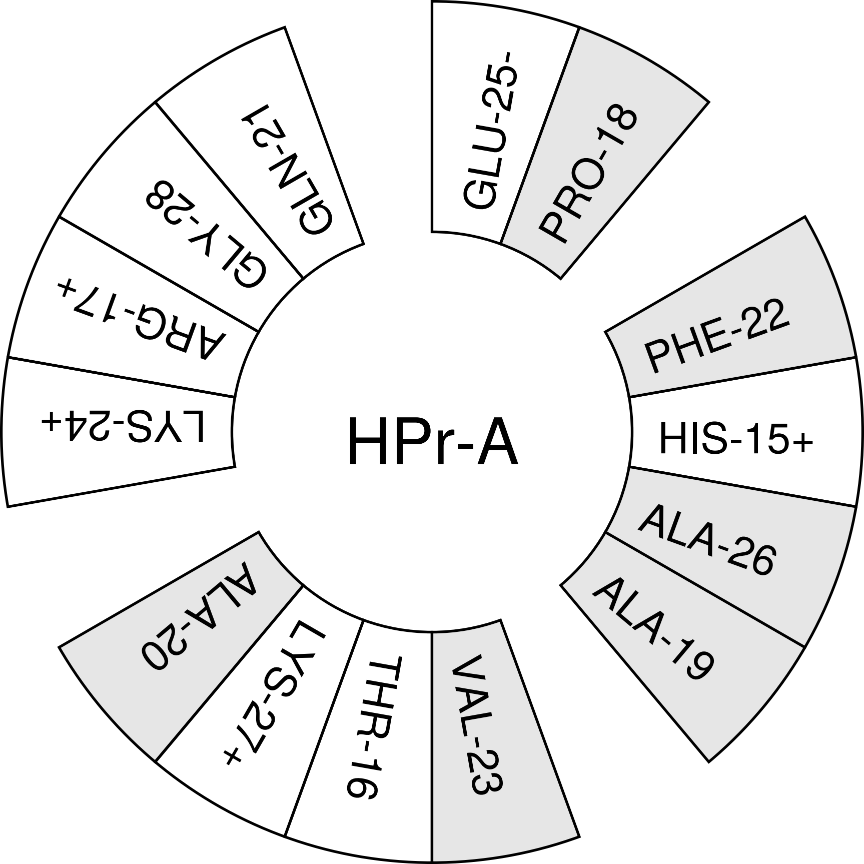
HPr-A
GLU-25-
PRO-18
PHE-22
HIS-15+
ALA-26
ALA-19
VAL-23
THR-16
LYS-27+
ALA-20
LYS-24+
ARG-17+
GLY-28
GLN-21
