# Supplementary material for: gmxapi: A GROMACS-native Python interface for molecular dynamics with ensemble and plugin support
Source: PLoS Comput Biol. 2022 Feb 14;18(2):e1009835. doi: 10.1371/journal.pcbi.1009835 (PMC8880871; doi:10.1371/journal.pcbi.1009835)
Supplement: S1 Software Archive — (TGZ) [file pcbi.1009835.s002.tgz › 2022-software/gromacs-v2022-beta2.tar.bz2!/gromacs-v2022-beta2/docs/reference-manual/analysis/plots/msdwater.pdf]

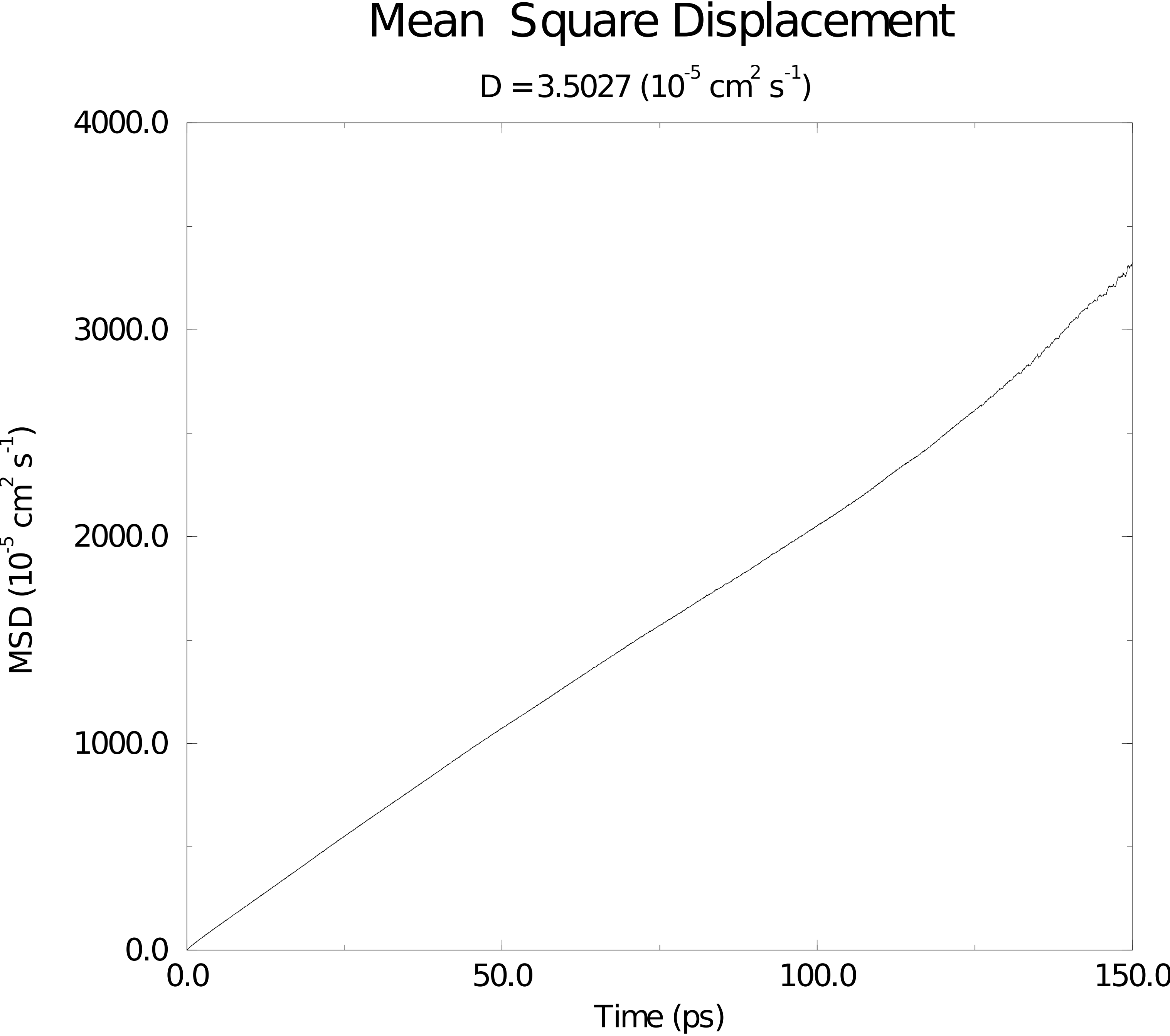

Mean Square Displacement
D = 3.5027 (10-5 cm2 s-1)
4000.0
3000.0
2000.0
1000.0
0.0
MSD (10-5 cm2 s-1)
0.0
50.0
100.0
150.0
Time (ps)
