# Supplementary material for: gmxapi: A GROMACS-native Python interface for molecular dynamics with ensemble and plugin support
Source: PLoS Comput Biol. 2022 Feb 14;18(2):e1009835. doi: 10.1371/journal.pcbi.1009835 (PMC8880871; doi:10.1371/journal.pcbi.1009835)
Supplement: S1 Software Archive — (TGZ) [file pcbi.1009835.s002.tgz › 2022-software/gromacs-v2022-beta2.tar.bz2!/gromacs-v2022-beta2/docs/reference-manual/functions/plots/fig-02.pdf]

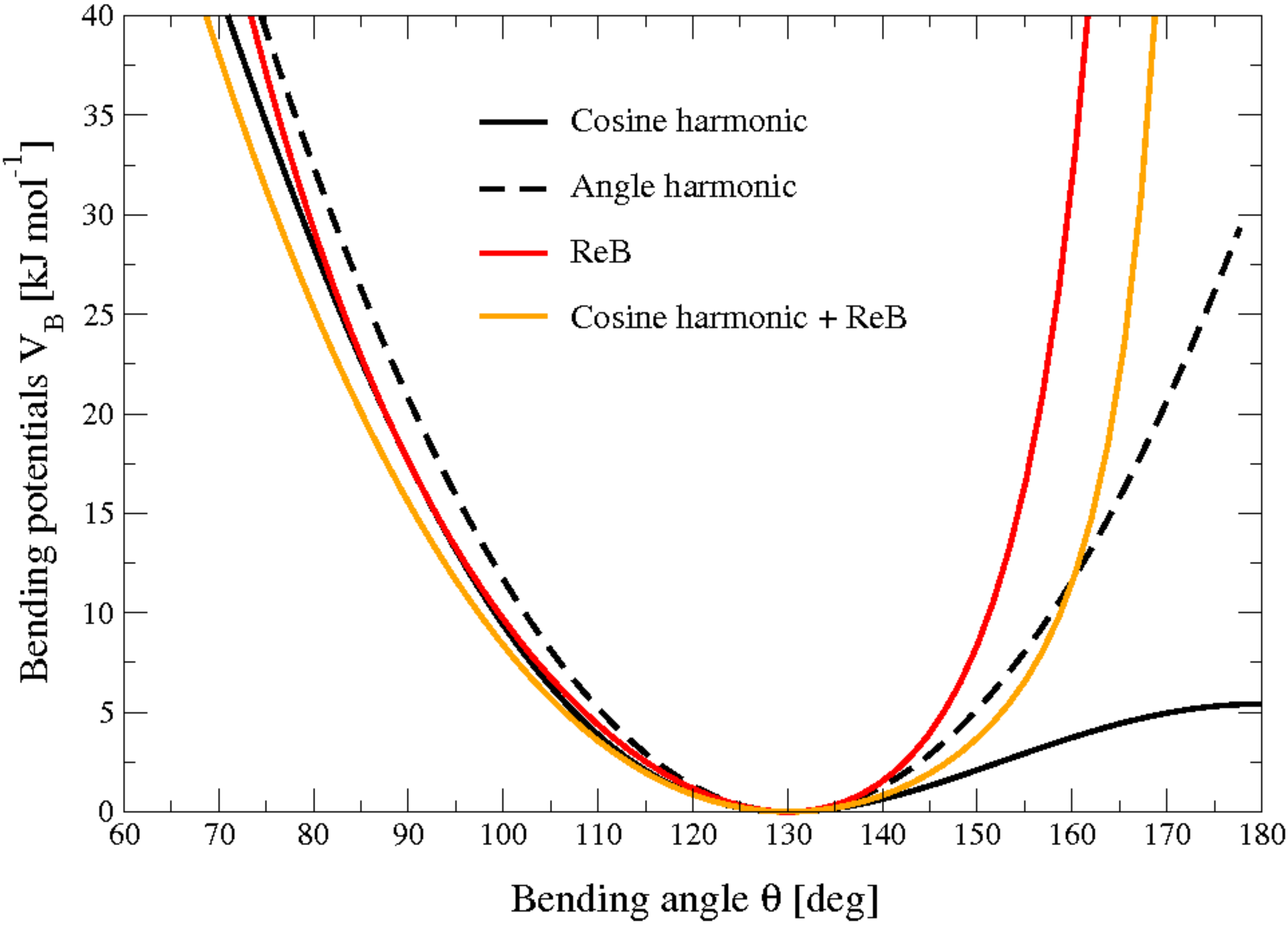

Cosine harmonic
Angle harmonic
ReB
Cosine harmonic + ReB
Bending potentials V_B [kJ mol^-1]
Bending angle θ [deg]
0
5
10
15
20
25
30
35
40
60
70
80
90
100
110
120
130
140
150
160
170
180
