# Supplementary material for: gmxapi: A GROMACS-native Python interface for molecular dynamics with ensemble and plugin support
Source: PLoS Comput Biol. 2022 Feb 14;18(2):e1009835. doi: 10.1371/journal.pcbi.1009835 (PMC8880871; doi:10.1371/journal.pcbi.1009835)
Supplement: S1 Software Archive — (TGZ) [file pcbi.1009835.s002.tgz › 2022-software/gromacs-v2022-beta2.tar.bz2!/gromacs-v2022-beta2/docs/reference-manual/functions/plots/gapsys-sc.pdf]

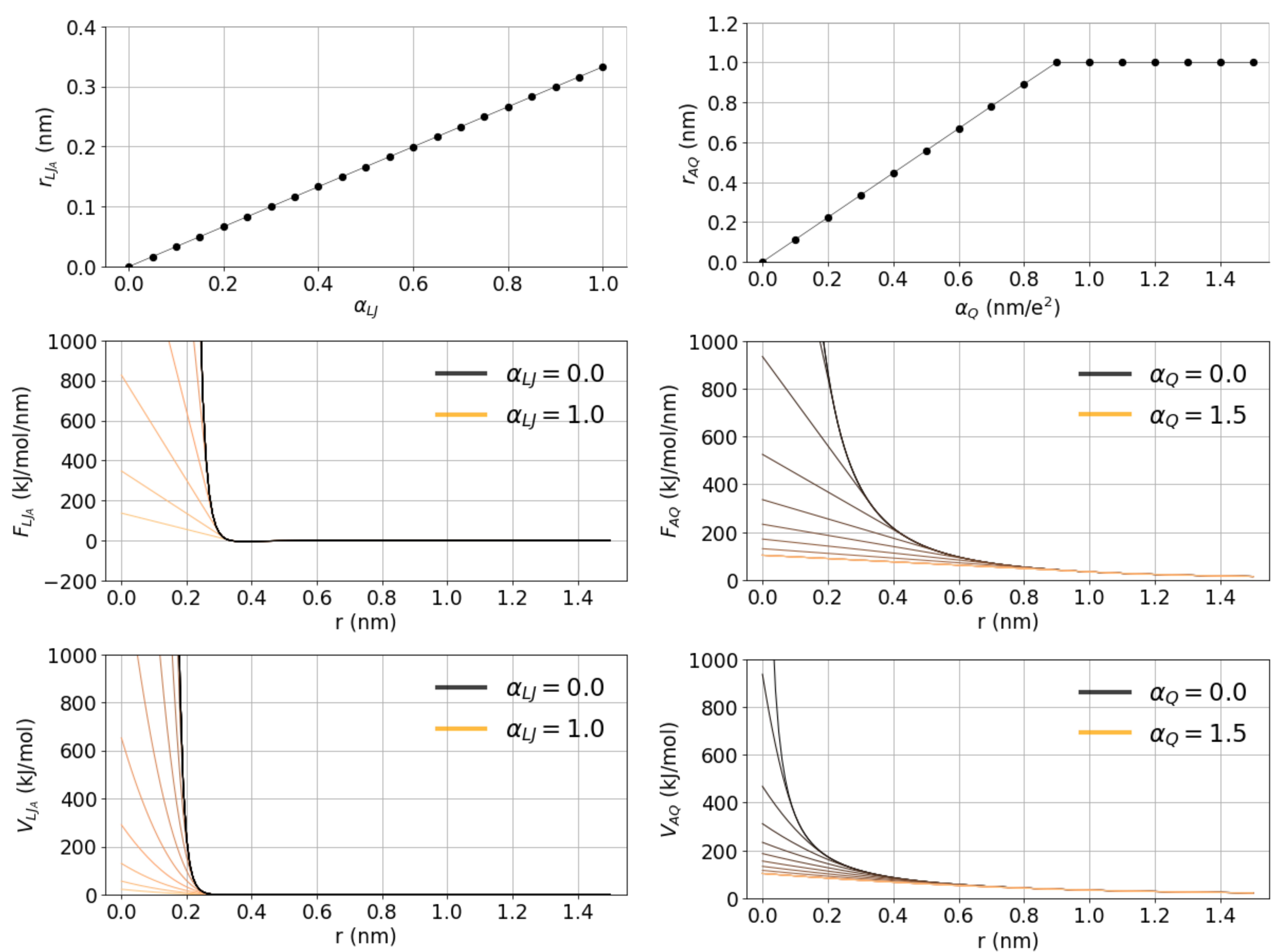

r_{LJ_A} (nm)
α_{LJ}
r_{AQ} (nm)
α_Q (nm/e^2)
F_{LJ_A} (kJ/mol/nm)
r (nm)
$\alpha_{LJ} = 0.0$
$\alpha_{LJ} = 1.0$
F_{AQ} (kJ/mol/nm)
r (nm)
$\alpha_Q = 0.0$
$\alpha_Q = 1.5$
V_{LJ_A} (kJ/mol)
r (nm)
$\alpha_{LJ} = 0.0$
$\alpha_{LJ} = 1.0$
V_{AQ} (kJ/mol)
r (nm)
$\alpha_Q = 0.0$
$\alpha_Q = 1.5$
