# Supplementary material for: gmxapi: A GROMACS-native Python interface for molecular dynamics with ensemble and plugin support
Source: PLoS Comput Biol. 2022 Feb 14;18(2):e1009835. doi: 10.1371/journal.pcbi.1009835 (PMC8880871; doi:10.1371/journal.pcbi.1009835)
Supplement: S1 Software Archive — (TGZ) [file pcbi.1009835.s002.tgz › 2022-software/gromacs-v2022-beta2.tar.bz2!/gromacs-v2022-beta2/docs/reference-manual/plots/drift-all.pdf]

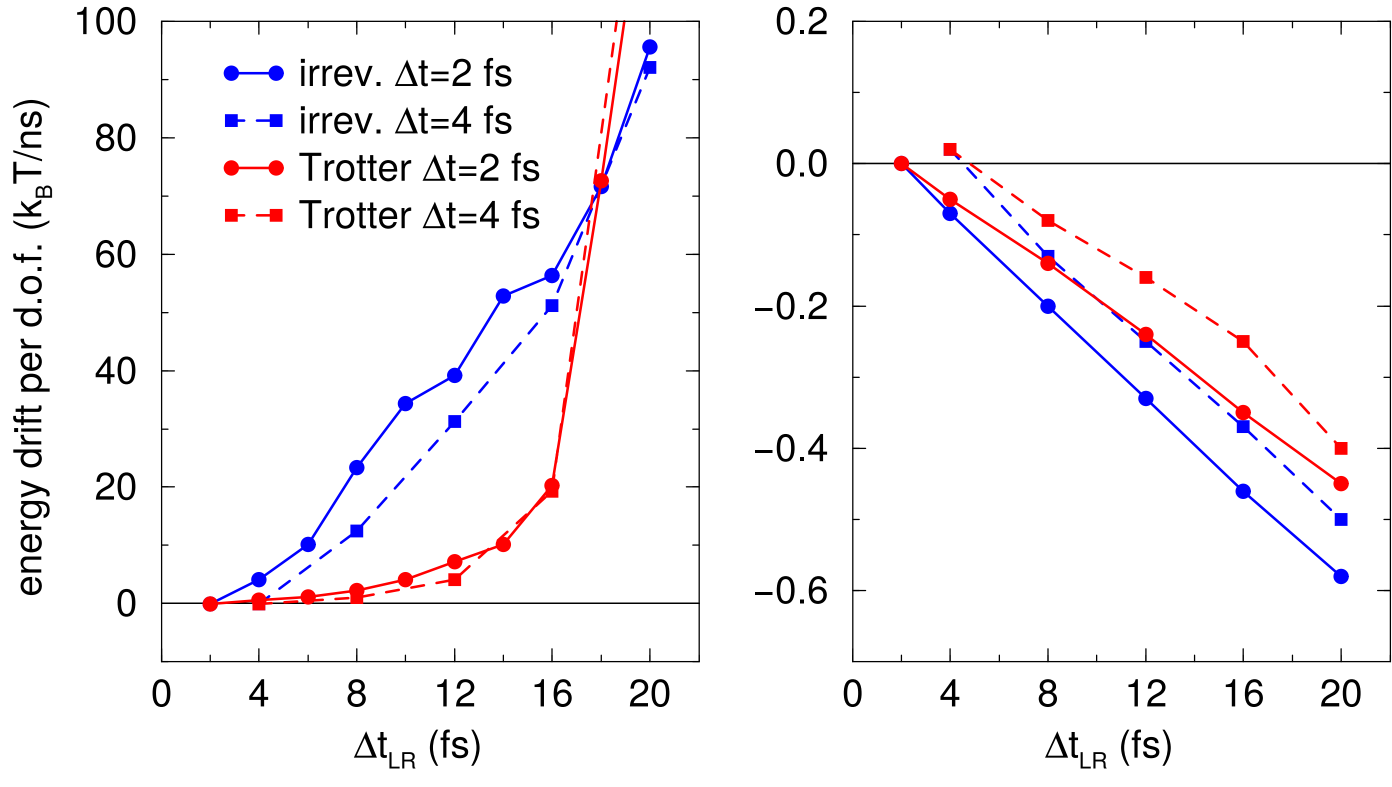

irrev. Δt=2 fs
irrev. Δt=4 fs
Trotter Δt=2 fs
Trotter Δt=4 fs
energy drift per d.o.f. (k_B T/ns)
Δt_LR (fs)
Δt_LR (fs)
0
20
40
60
80
100
0
4
8
12
16
20
0.2
0.0
−0.2
−0.4
−0.6
