# Supplementary material for: gmxapi: A GROMACS-native Python interface for molecular dynamics with ensemble and plugin support
Source: PLoS Comput Biol. 2022 Feb 14;18(2):e1009835. doi: 10.1371/journal.pcbi.1009835 (PMC8880871; doi:10.1371/journal.pcbi.1009835)
Supplement: S1 Software Archive — (TGZ) [file pcbi.1009835.s002.tgz › 2022-software/gromacs-v2022-beta2.tar.bz2!/gromacs-v2022-beta2/docs/reference-manual/plots/ring.pdf]

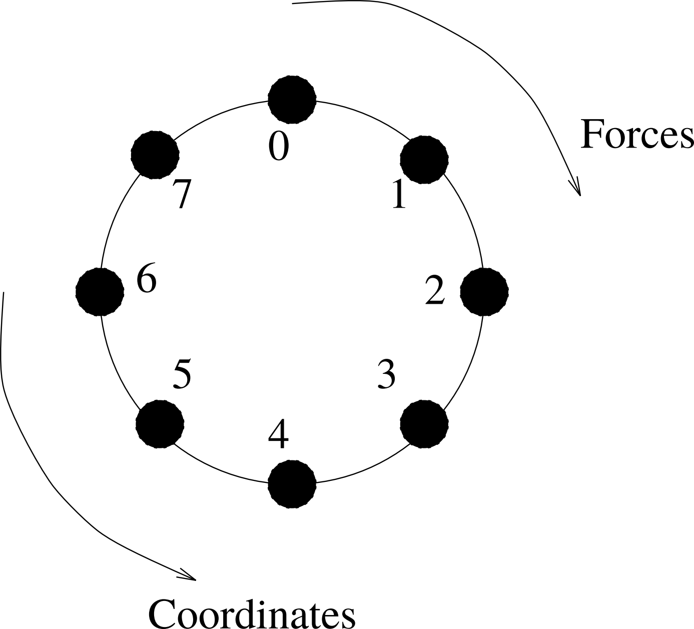
Forces
0
1
2
3
4
5
6
7
Coordinates
