# Supplementary material for: gmxapi: A GROMACS-native Python interface for molecular dynamics with ensemble and plugin support
Source: PLoS Comput Biol. 2022 Feb 14;18(2):e1009835. doi: 10.1371/journal.pcbi.1009835 (PMC8880871; doi:10.1371/journal.pcbi.1009835)
Supplement: S1 Software Archive — (TGZ) [file pcbi.1009835.s002.tgz › 2022-software/gromacs-v2022-beta2.tar.bz2!/gromacs-v2022-beta2/docs/reference-manual/special/plots/equipotential.pdf]

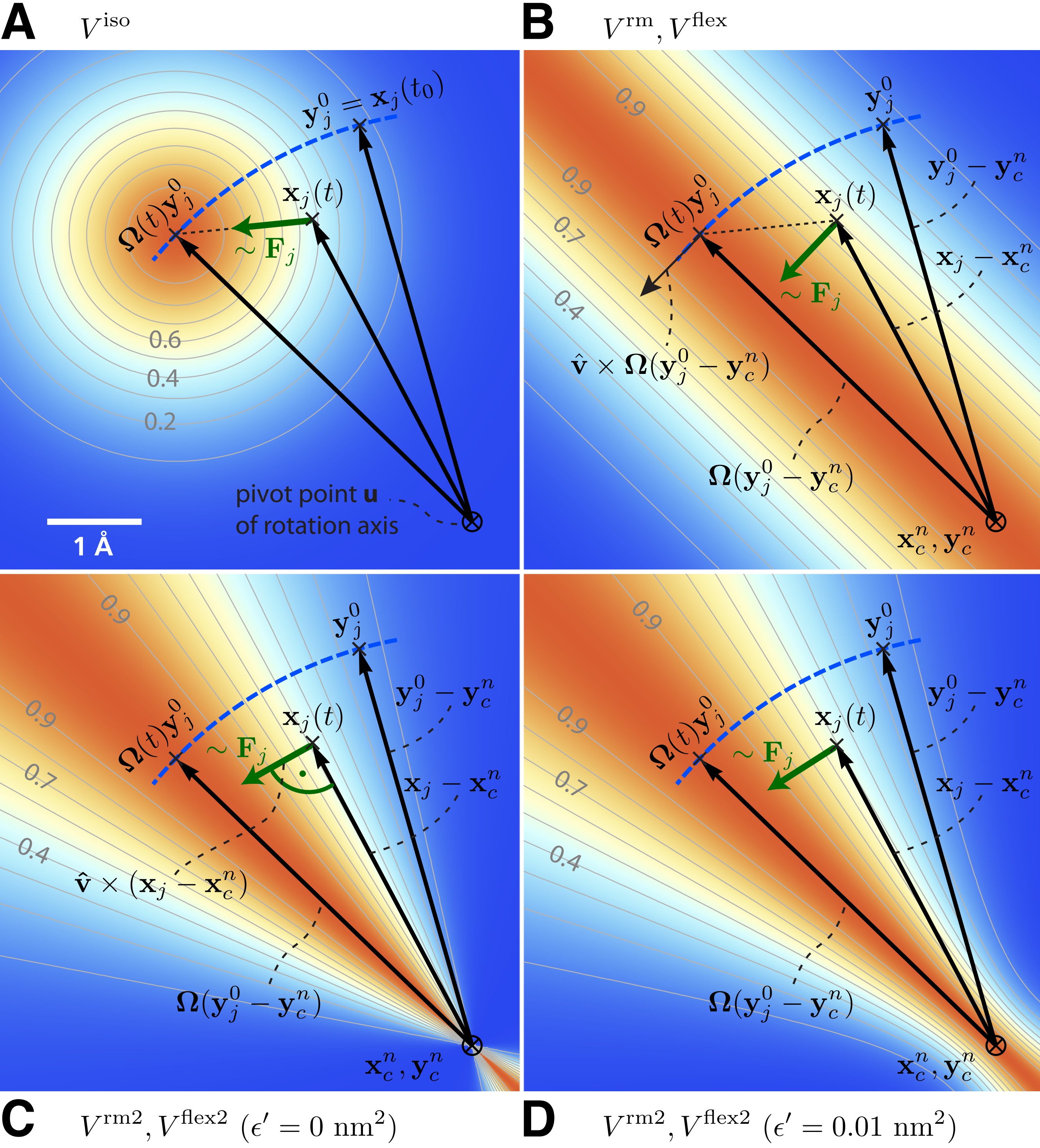
A
V^{iso}
$\mathbf{y}_j^0 = \mathbf{x}_j(t_0)$
$\mathbf{x}_j(t)$
$\mathbf{\Omega}(t)\mathbf{y}_j^0$
$\sim \mathbf{F}_j$
0.6
0.4
0.2
pivot point **u**
of rotation axis
1 Å
B
$V^{\mathrm{rm}}, V^{\mathrm{flex}}$
0.9
0.9
0.7
0.4
$\mathbf{y}_j^0$
$\mathbf{y}_j^0 - \mathbf{y}_c^n$
$\mathbf{x}_j(t)$
$\mathbf{\Omega}(t)\mathbf{y}_j^0$
$\mathbf{x}_j - \mathbf{x}_c^n$
$\sim \mathbf{F}_j$
$\hat{\mathbf{v}} \times \mathbf{\Omega}(\mathbf{y}_j^0 - \mathbf{y}_c^n)$
$\mathbf{\Omega}(\mathbf{y}_j^0 - \mathbf{y}_c^n)$
$\mathbf{x}_c^n, \mathbf{y}_c^n$
0.9
0.9
0.7
0.4
$\mathbf{y}_j^0$
$\mathbf{y}_j^0 - \mathbf{y}_c^n$
$\mathbf{x}_j(t)$
$\mathbf{\Omega}(t)\mathbf{y}_j^0$
$\sim \mathbf{F}_j$
$\mathbf{x}_j - \mathbf{x}_c^n$
$\hat{\mathbf{v}} \times (\mathbf{x}_j - \mathbf{x}_c^n)$
$\mathbf{\Omega}(\mathbf{y}_j^0 - \mathbf{y}_c^n)$
$\mathbf{x}_c^n, \mathbf{y}_c^n$
0.9
0.9
0.7
0.4
$\mathbf{y}_j^0$
$\mathbf{y}_j^0 - \mathbf{y}_c^n$
$\mathbf{x}_j(t)$
$\mathbf{\Omega}(t)\mathbf{y}_j^0$
$\sim \mathbf{F}_j$
$\mathbf{x}_j - \mathbf{x}_c^n$
$\mathbf{\Omega}(\mathbf{y}_j^0 - \mathbf{y}_c^n)$
$\mathbf{x}_c^n, \mathbf{y}_c^n$
C
$V^{\mathrm{rm2}}, V^{\mathrm{flex2}}$ ($\epsilon' = 0$ nm^2)
D
$V^{\mathrm{rm2}}, V^{\mathrm{flex2}}$ ($\epsilon' = 0.01$ nm^2)
