# Supplementary material for: gmxapi: A GROMACS-native Python interface for molecular dynamics with ensemble and plugin support
Source: PLoS Comput Biol. 2022 Feb 14;18(2):e1009835. doi: 10.1371/journal.pcbi.1009835 (PMC8880871; doi:10.1371/journal.pcbi.1009835)
Supplement: S1 Software Archive — (TGZ) [file pcbi.1009835.s002.tgz › 2022-software/gromacs-v2022-beta2.tar.bz2!/gromacs-v2022-beta2/docs/reference-manual/special/plots/gaussians.pdf]

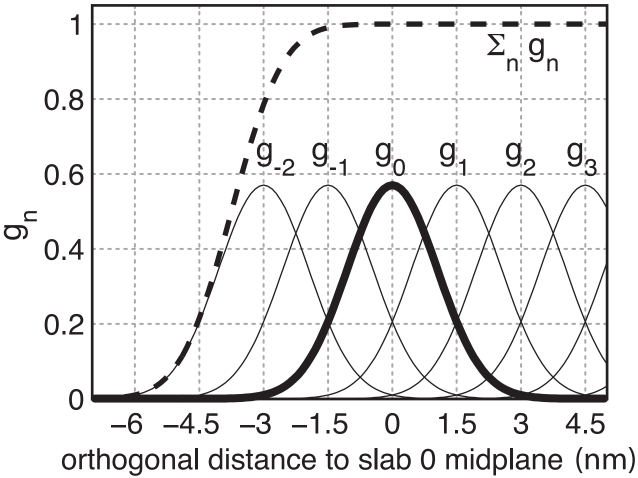
1
0.8
0.6
0.4
0.2
0
g_n
$\Sigma_n g_n$
g_{-2}
g_{-1}
g_0
g_1
g_2
g_3
−6
−4.5
−3
−1.5
0
1.5
3
4.5
orthogonal distance to slab 0 midplane (nm)
