# Supplementary material for: gmxapi: A GROMACS-native Python interface for molecular dynamics with ensemble and plugin support
Source: PLoS Comput Biol. 2022 Feb 14;18(2):e1009835. doi: 10.1371/journal.pcbi.1009835 (PMC8880871; doi:10.1371/journal.pcbi.1009835)
Supplement: S1 Software Archive — (TGZ) [file pcbi.1009835.s002.tgz › 2022-software/gromacs-v2022-beta2.tar.bz2!/gromacs-v2022-beta2/src/external/googletest/docs/_layouts/default.html]

{% seo %}


# {{ site.title | default: "Documentation" }}


{% for item in site.data.navigation.nav %}

## {{ item.section }}

{% for subitem in item.items %}
- {{ subitem.title }}
{% endfor %}
{% endfor %}

{{ content }}

GoogleTest ·
GitHub Repository ·
License ·
Privacy Policy
